# Integrating Deoxyribozymes into Colorimetric Sensing Platforms

**DOI:** 10.3390/s16122061

**Published:** 2016-12-03

**Authors:** Dingran Chang, Sandy Zakaria, Mimi Deng, Nicholas Allen, Kha Tram, Yingfu Li

**Affiliations:** 1 Department of Biochemistry and Biomedical Sciences, McMaster University, Hamilton, ON L8S 4K1, Canada; changd3@mcmaster.ca (D.C.); dengxx3@mcmaster.ca (M.D.); allenn5@mcmaster.ca (N.A.); tramkq@mcmaster.ca (K.T.); 2 Department of Biomedical Engineering, McMaster University, Hamilton, ON L8S 4K1, Canada; zakarias@mcmaster.ca

**Keywords:** DNAzymes, biosensors, colorimetric sensors

## Abstract

Biosensors are analytical devices that have found a variety of applications in medical diagnostics, food quality control, environmental monitoring and biodefense. In recent years, functional nucleic acids, such as aptamers and nucleic acid enzymes, have shown great potential in biosensor development due to their excellent ability in target recognition and catalysis. Deoxyribozymes (or DNAzymes) are single-stranded DNA molecules with catalytic activity and can be isolated to recognize a wide range of analytes through the process of in vitro selection. By using various signal transduction mechanisms, DNAzymes can be engineered into fluorescent, colorimetric, electrochemical and chemiluminescent biosensors. Among them, colorimetric sensors represent an attractive option as the signal can be easily detected by the naked eye. This reduces reliance on complex and expensive equipment. In this review, we will discuss the recent progress in the development of colorimetric biosensors that make use of DNAzymes and the prospect of employing these sensors in a range of chemical and biological applications.

## 1. A Brief Outlook of Biosensors

Biosensors are analytical devices that utilize a biological component for the detection of a specific analyte [1]. This idea was first conceptualized in 1962 by Leland Clark with the development of an enzyme-based biosensor that could monitor blood glucose levels [2]. This biosensor, the first and perhaps the most widely known example, uses glucose oxidase to generate an electrochemical signal, which is quantified through the use of an oxygen electrode. This system was further developed into the modern-day glucose meter, a device which has become a staple element in the treatment and management of diabetes mellitus, and to this day is the largest stakeholder in the biosensor market [3,4]. Since Clark’s invention, interest in biosensors has grown for uses ranging from sensitive laboratory analysis to point-of-care devices.

As the development of biosensors accelerated, two broad categories of biosensors emerged: simple and complex. Simple biosensors, such as the aforementioned glucose monitor, tend to sacrifice sensitivity and throughput with the goal of greatly reducing cost, complexity and size, while remaining functional when faced with the chemical and biological variability of real world samples. Conversely, complex biosensors such as immunosensors are designed for laboratory use, where factors such as sensitivity, applicability to specialized usage scenarios, and ability to process large amounts of samples are far more important than ease-of-use and cost.

**Figure 1:**
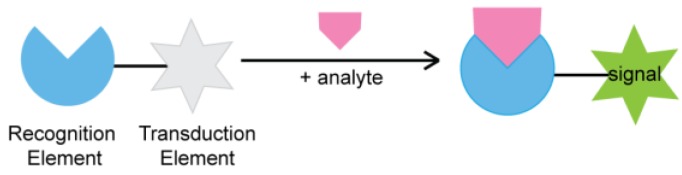
Schematic representation of biosensors.

Although the field of biosensors has seen a vast expansion over the last 50 years, the core concept of a biosensor, as shown in Figure 1, remains the same. Every biosensor is composed of a recognition element and a transduction element. The types of recognition elements used, originally limited to protein enzymes and antibodies, now include nucleic acids, bacterial cells, and even whole tissues, increasing the variety and complexity of analytes that can be detected [5,6,7]. Transduction elements in biosensors have also seen considerable innovation, particularly in expanding the range of generated response signals. Originally limited to electric signals, modern-day biosensors feature an assortment of output signal types, such as fluorescent, optical, and thermal signals [8,9,10,11].

### 1.1. The Next Generation of Recognition Elements

To date, the majority of biosensors on the market use protein enzymes or antibodies as their recognition element, and development of new biosensors continues to strongly focus on this model [12]. While proteins have proven to be capable in this task, their use as a recognition element comes with several limitations. Protein enzymes are naturally evolved catalysts that are limited to a target ligand. As a result, they offer little flexibility for developing biosensors to detect analytes that do not have a known natural enzyme that binds to them. While antibodies are more flexible in this regard (i.e., they can be developed for an arbitrary target), antibody isolation is costly and difficult to scale up for mass production [13].

In response to these limitations of proteins, the last decade has seen increasing research into the use of functional nucleic acids (FNAs) as biosensors, and in biotechnology in general as alternatives to proteins in a variety of uses [4]. FNAs are similar to proteins in function and purpose, but they are composed of DNA and RNA. FNA-based catalysts, known as DNAzymes (when composed of DNA) or ribozymes (when composed of RNA), can perform catalytic functions in the same vein as protein enzymes. On the other hand, aptamers are DNA or RNA molecules capable of binding to a specific target, making them analogous to antibodies in function [14]. In particular, the bulk of research on FNAs has focused on DNAzymes and DNA aptamers, due to their greatly increased stability compared to their RNA counterparts.

### 1.2. Using Functional Nucleic Acids for Developing Biosensors

**Figure 2:**
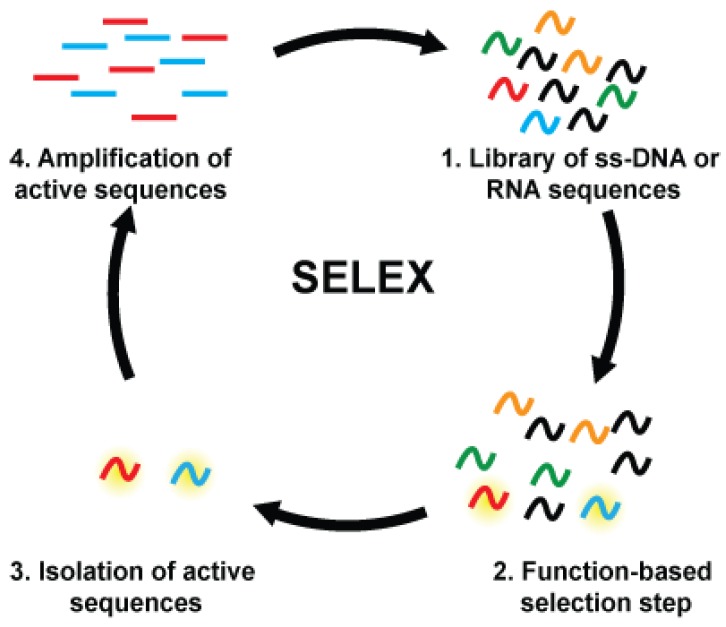
A general in vitro selection approach for isolating functional nucleic acids.

In contrast to protein enzymes and antibodies both of which exist in nature, all DNAzymes and DNA aptamers are developed artificially through a technique known as in vitro selection, or SELEX (Systematic Evolution of Ligands by Exponential Enrichment). In vitro selection is a combinatorial approach that subjects a randomized library of 10^14^–10^16^ DNA or RNA molecules to successive rounds of selection and amplification in which the sequences expressing the desired function are enriched and inactive sequences are discarded (Figure 2) [15,16,17]. This allows the development of fully synthetic aptamers or DNAzymes that perform an arbitrary function. The development of the in vitro selection technique allows for the discovery of a great array of man-made FNAs that could detect a wide range of targets, from small molecules to proteins to whole cells [18]. 

FNAs have multiple advantages over proteins for use in biosensors. Firstly, FNAs can be isolated through in vitro selection to detect essentially any target of choice including those for which antibodies are difficult to obtain, such as toxic metal ions and molecules with poor immunogenicity. Secondly, FNAs are chemically synthesized, rather than expressed in biological systems. This makes FNA production much more commercially scalable, increases consistency between batches, and eliminates biological contamination from expression hosts. Moreover, FNAs can be easily chemically modified in order to increase stability, as well as to introduce functional groups to allow for conjugation chemistry, enabling the linking of FNAs to small molecules (dyes, carbohydrates, amino acids), proteins, and various nanomaterials. Lastly, FNAs are resistant to thermal denaturation, and are able to re-fold into their native structures after heating, a property most proteins lack. Owing to these advantages, FNAs have been extensively used as the recognition element and signal amplifier for biosensing applications [18,19,20,21,22,23].

Although FNAs have been applied in a variety of ways to create new biosensors [18,19,20,21,22,23], this review paper will focus specifically on colorimetric DNAzyme-based biosensors, due to their promising potential for applications in point-of-case devices. DNAzymes can be integrated with various colorimetric signal transduction elements, such as nanoparticles, enzymes, and organic dyes, to achieve colorimetric biosensing. Here, the recent development of different colorimetric signal transducers applied in DNAzyme-based biosensing will be discussed.

## 2. Gold Nanoparticles as Colorimetric Signal Transducer

In nanotechnology, gold nanoparticles (AuNPs) are the basis of many applications due to their plasmonic effects, high stability, and electronic properties [24,25].The intrinsic properties of AuNPs are dependent on AuNPs’ size, shape and surface structure [25,26,27]. The wide-ranging functionalities of AuNPs are recognized in the development of biosensors [28,29,30,31], nanoassembly [32,33,34,35,36,37,38,39], biolabeling of molecules and drug delivery [40,41,42]. The prominent role of AuNPs for the development of robust colorimetric sensors will be discussed in this section. 

Over the last two decades, AuNPs have been used as the nanoassembly units for creating colorimetric biosensors. AuNPs’ intrinsic properties, namely surface plasmon resonance (SPR) and colloidal stability are contributing factors for a colorimetric generation. SPR is the mechanism by which AuNPs can produce visible light in response to an electromagnetic field. When AuNPs are exposed to light, the resonant wavelength is absorbed by the AuNPs to induce the oscillation of their surface electrons [24,25]. These oscillating electrons will produce electromagnetic radiation that can be observed by the naked eye. Colloid AuNPs are stabilized by two forces counterbalancing one another: van der Waals and electrostatic forces [43]. When one of these forces is weak or absent, the colloid system will destabilize and the AuNPs will aggregate, resulting in a color shift from red to blue (the shift of the surface plasmon band to a longer wavelength). This is known as interparticle plasmon coupling and has been extensively used in colorimetric biosensor development [44]. Currently, techniques such as using colloidal stabilizers and cross-linking agents have shown to be successful in manipulating the dispersal and aggregation AuNPs for colorimetric generation. These techniques, along with others, are listed in Table 1.

Due to DNA’s programmable nature, the functionalization of AuNPs with DNA molecules provides an attractive option for selective colorimetric sensing. AuNPs are generally coated by ligands during synthesis to stabilize the colloid aqueous solutions [59,60,61]. These ligands on the surface of AuNPs can be efficiently replaced by thiol-containing molecules because of the thiophilic nature of Au [47,62]. Based on this principle, thiol-modified DNAs (thiol-DNAs) can be successfully functionalized onto the surface of AuNPs for further biosensing application. In addition to thiol-DNA, unmodified single-stranded DNA (ssDNA) was found to bind AuNPs with sufficient affinity, while double-stranded DNA (dsDNA) could not [63,64,65]. By introducing DNAzymes as recognition elements into AuNP systems, a series of colorimetric biosensors has been developed for a broad range of targets [44,66]. Here, we will present an overview of the signaling strategies of DNAzyme-functionalized AuNPs. 

### 2.1. Signaling Using Unlabeled Gold Nanoparticles (AuNPs) and Deoxyribozymes (DNAzymes)

**Figure 3:**
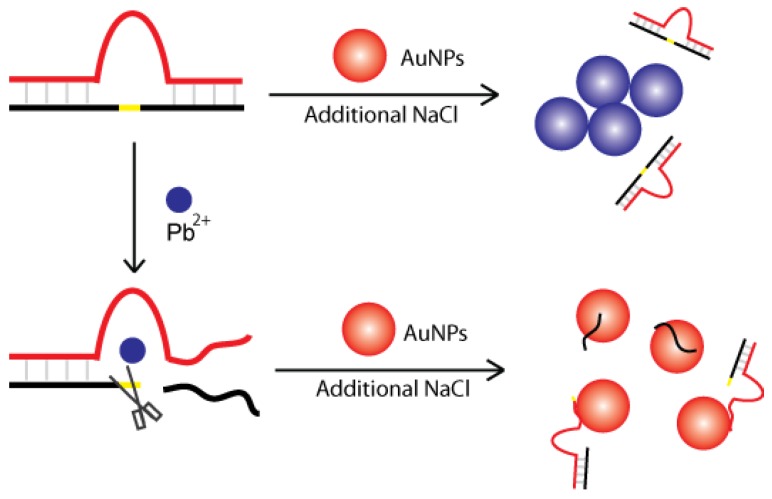
Label-free detection of Pb^2+^ using the 8–17 DNAzyme and AuNPs.

High salt concentrations increase electrostatic interactions and reduce colloid stability. This will result in the aggregation of AuNPs and a color shift from red to blue. However, this can be prevented by the addition of ssDNA which can be adsorbed onto the surface of unmodified AuNPs, thus protecting the AuNPs from aggregation. Based on this, label-free detection of Pb^2+^ has been demonstrated by coupling Pb^2+^-dependent DNAzyme with AuNPs [67,68]. Upon cleavage of the nucleic acid substrate by an 8–17 DNAzyme in the presence of Pb^2+^, an ssDNA fragment was released which could then be bound to the AuNPs’ surface (Figure 3). When additional salt was added, AuNPs remained dispersed in solution due to enhanced stability, resulting in no color change. Conversely, without the presence of Pb^2+^, ssDNA was not produced to protect AuNPs from aggregation and a color shift was observed. This technique can achieve low Pb^2+^ detection limits of 3 nM. Similar designs have been reported using different nucleic acid-cleaving DNAzymes for detecting Cu^2+^ [69] and UO^2+^ [70].

### 2.2. Signaling by Assembling or Disassembling Cross-Linked AuNPs Using DNAzymes

**Figure 4:**
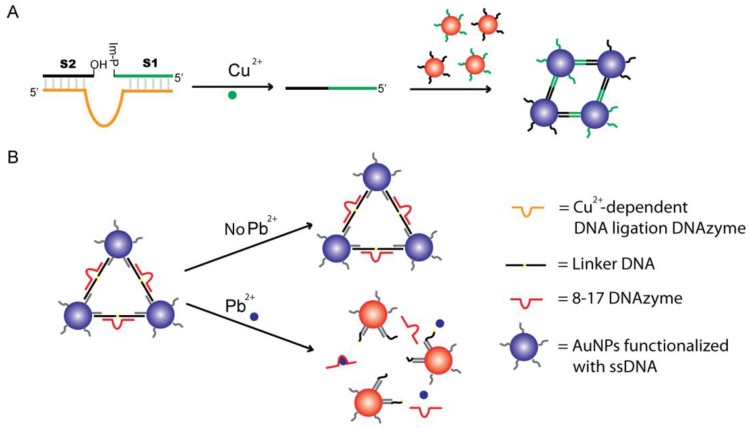
DNAzyme-directed AuNP assemblies as biosensors for (**A**) Cu^2+^; and (**B**) Pb^2+^.

When AuNPs are functionalized with thiol-modified DNA, the distance between AuNPs can be controlled by a linking DNA that is complementary to the DNA immobilized on AuNPs. As shown by Lu and colleagues, a DNA linker can hybridize with short DNA sequences on each AuNP to create a cross-linked network of AuNPs and induces aggregation [71]. In the presence of Cu^2+^, the Cu^2+^-dependent DNA ligation DNAzyme catalyzes the nucleophilic attack of the phosphorus center of substrate S1 by the hydroxyl group on substrate S2, forming a phosphodiester bond with the imidazole acting as a leaving group. The ligation product was designed as a DNA linker to assemble AuNPs and lead to a color shift from red to blue, as depicted in Figure 4A.

An alternate design presented by Lu and colleagues uses RNA-cleaving DNAzymes to disassemble cross-linked AuNPs [28,72]. In the absence of Pb^2+^, the DNAzyme remains bound to the AuNPs and no color shift is observed. However, when Pb^2+^ is added to the solution, the DNAzyme will respond by cleaving the DNA cross-linker, separating the AuNP complex. This will result in the dispersal of AuNPs and a color shift from blue to red will be observed, as outlined by Figure 4B. Similar designs have been used in detecting UO^2+^ [56], adenosine [73] and infectious pathogens [74].

### 2.3. G-Quadruplex DNAzyme-Controlled Aggregation of AuNPs 

**Figure 5:**
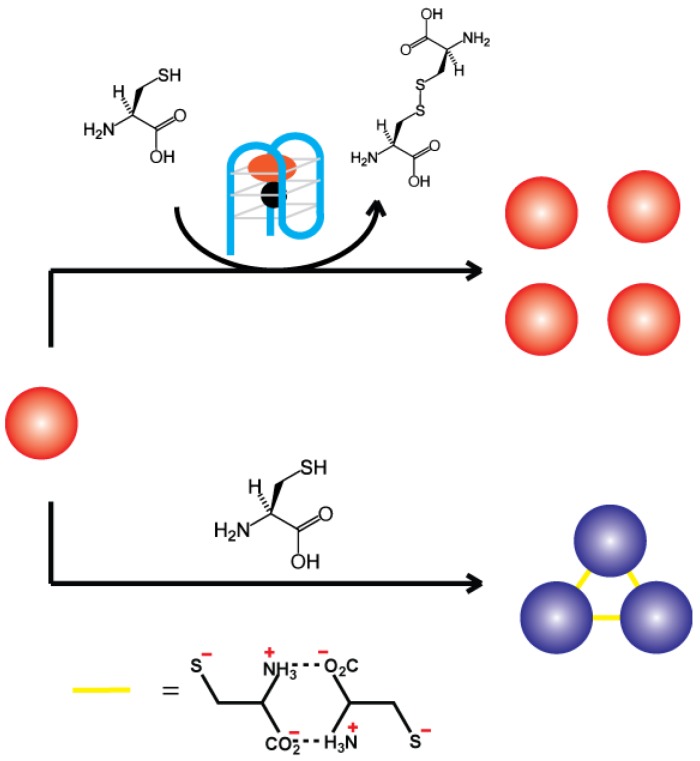
Controlling AuNP aggregation by regulating cysteine oxidation using a G-quadruplex DNAzyme.

Recently, Willner and coworkers reported a sensing platform by integrating a G-quadruplex DNAzyme with AuNPs [75]. As shown in Figure 5, the thiol groups found in l-cysteine were able to bind to AuNPs to induce aggregation, resulting in a color transition from red to blue. However, a hemin/G-quadruplex peroxidase-mimicking DNAzyme could oxidize these thiol groups into disulfides, thereby converting l-cysteine to cystine, a process that inhibits the aggregation of AuNPs. The degree of aggregation inhibition was controlled by the concentration of the DNAzyme in the system. These functions were implemented for ssDNA detection. The sensing platform composed of a hairpin DNA structure that includes a recognition site for the DNA target and an inactivated caged G-quadruplex sequence. Upon binding of the DNA target to the complex, the G-quadruplex is released and activated. This sensing platform has also been applied for the analysis of aptamer-substrate complexes, and for the analysis of l-cysteine in human urine samples [75].

### 2.4. AuNPs with Other Techniques

In addition to manipulating dispersal and aggregation AuNPs for color change, the red AuNPs can be directly used as a signal indicator together with other techniques such as graphene oxide, hydrogel and lateral-flow devices for biosensing.

**Figure 6:**
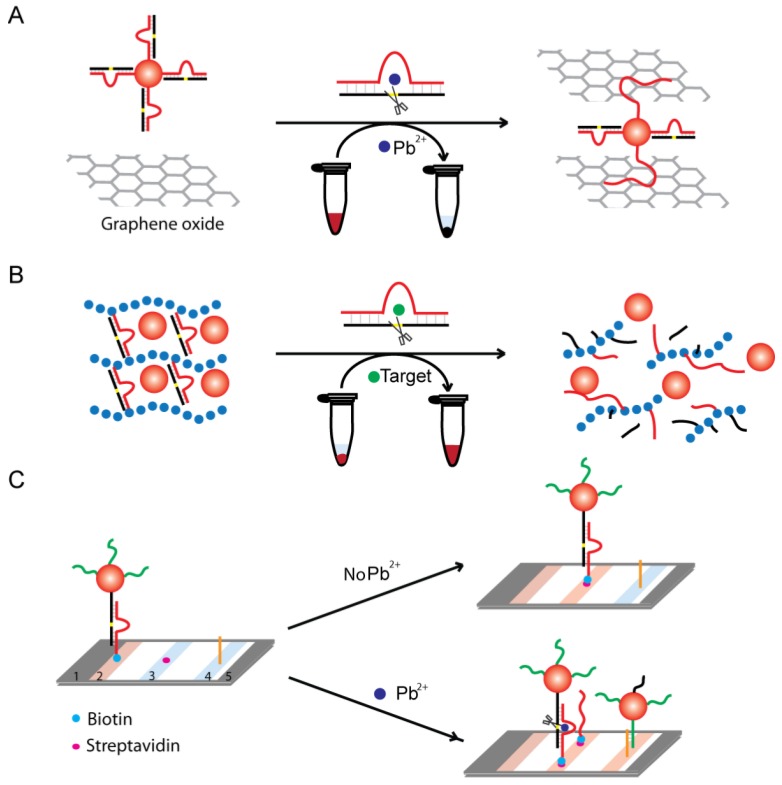
(**A**) Using DNAzyme-functionalized AuNPs and graphene oxide for Pb^2+^ detection; (**B**) Hydrogel-sensing platform with DNAzyme-functionalized AuNPs; (**C**) Lateral-flow device using DNAzyme-functionalized AuNPs for Pb^2+^ detection. Structure of lateral-flow device—1: Wicking pad, 2: Conjugation pad, 3: Control zone, 4: Test zone, 5: Absorption pad.

Graphene oxide, a one-atom-thick 2D nanomaterial, is capable of binding ssDNA strongly, but not double-strand DNA. Recently, Li and coworkers developed a “red to white” biosensor for Pb^2+^ by integrating 8–17 DNAzyme functionalized AuNPs with graphene oxide [76] as shown in Figure 6A. The system begins with a DNA duplex that contains the RNA substrate and DNAzyme hybridized together. This duplex is then conjugated onto the AuNP’s surface. In the absence of Pb^2+^, the DNAzyme duplex has a low binding affinity to graphene oxide leading to a diffused red color seen throughout the solution. However, in the presence of Pb^2+^, the DNAzyme cleaves the substrate strand, resulting in the disassembly of the DNAzyme duplex forming an extended single-stranded moiety that has a much higher affinity towards graphene oxide. This entrapment of ssDNA-AuNPs removes freely diffused AuNPs and causes the solution to become colorless.

Hydrogels are soft materials with a cross-linked 3D network structure that contains a large fraction of water within their structure. In a hydrogel, the backbone polymers are held together by cross-linkers, which keep the chains in the polymer matrix together. Stimuli-responsive smart hydrogels have attracted particular attention in the development of biosensor devices with the advantages of simplicity, sensitivity, cost-effectiveness, and portability, as well as ease of storage [77,78]. In particular, DNA hydrogels with synthetic polymers being cross-linked by DNA have been engineered to create stable sensors. As shown in Figure 6B, the substrate-DNAzyme duplex is polymerized to the hydrogel. In addition, this highly cross-linked structure traps AuNPs within the hydrogel matrix. However, in the presence of a target, the DNAzyme is activated and cleaves the substrate sequence to destabilize the highly cross-linked hydrogel network resulting in the release of the caged AuNPs. The free AuNPs will be detected by the presence of red color seen throughout solution. Hydrogel colorimetric biosensors have been developed for ATP, cocaine, Pb^2+^ and Cu^2+^ [79,80,81].

User-friendly lateral-flow devices have also been developed based on DNAzymes and AuNPs for dipstick tests [82]. In one design, the paper-testing strips are separated into distinct zones with specialized functions: a conjugation pad, control zone, and test zone (Figure 6C). The conjugation pad contains the Pb^2+^-specific biotinylated DNAzyme-AuNPs complex. In the absence of Pb^2+^, the biotin-labeled complex migrated along the lateral-flow device and was captured by immobilized streptavidin located in the control zone. As a result, one red band was observed in the control zone as a sign of a Pb^2+^-free sample. However, when Pb^2+^ was present, the DNAzyme cleaved the biotin-tagged substrate from the AuNPs and allowed them to freely flow through the control zone to reach the test zone that contained an immobilized complementary DNA that captured the AuNPs to display a red-colored band. This technique has shown to be sensitive with a 20 nM detection limit and fast turnaround time of ten minutes for onsite polluted water samples.

## 3. Peroxidases as Colorimetric Signal Transducer

Peroxidases are a family of enzymes that catalyze the oxidation of a wide variety of substrates using H_2_O_2_ or other peroxides. They are widely used in analytical diagnostics, biotransformation, organic synthesis and treatment of waste waters [83]. The enzyme horseradish peroxidase (HRP) has the ability to produce a colored, fluorescent, or luminescent product when incubated with their respective substrates. As a result, it has been extensively used as a signal transducer in various techniques such as Western blotting and ELISA to determine the presence of a molecular target. H_2_O_2_-mediated oxidation of 3,3′,5,5′-tetramethylbenzidine (TMB) by HRP is a well-researched area of color-based sensing. HRP can provide colorimetric feedback on microscopic changes, making it an attractive feature in the field of functionalized nucleic acids. When used in conjunction with target-specific DNAzymes as recognition elements, HRP-based systems can report trace amounts of target.

**Figure 7:**
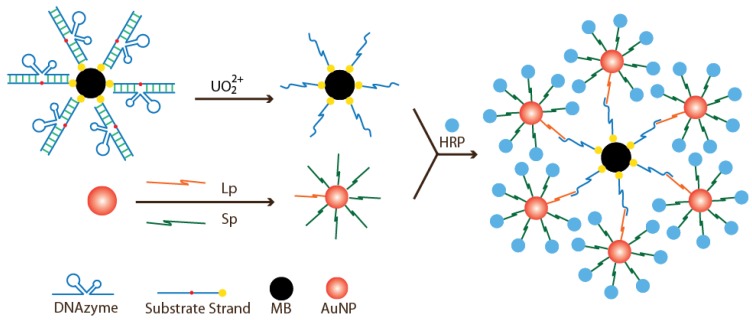
Experimental schematic of colorimetric UO_2_^2+^ detection using DNAzyme-conjugated MB for target recognition and HRP-functionalized AuNPs for signaling.

Zhang and coworkers recently reported a bioassay that used two structures: DNAzyme-functionalized magnetic beads (MB) for target recognition and AuNPs for catalytic signal amplification [84]. As shown in Figure 7, the first structure involves a biotin-modified substrate strand that is immobilized on streptavidin-coated magnetic beads and forms a duplex with a UO_2_^2+^-specific DNAzyme. The second structure involves conjugation of AuNPs to two thiol-modified DNA strands: (1) ligation probe; and (2) signal probe. In the presence of UO_2_^2+^, the substrate strand is cleaved, leaving behind a short single-strand DNA on the surface of MBs. This exposed short single-stranded DNA then hybridizes with the ligation probe that is attached to the AuNPs. Concurrently, the signal probe, which is modified with 3′-biotin captures the streptavidin-conjugated HRP to produce a super complex with many HRP molecules ready to catalyze a blue color generation upon TMB addition. The validity of the test was assessed using river water spiked with various UO_2_^2+^ levels determined by inductively coupled plasma mass spectrometry, a traditional method for analysis of trace metals in aqueous environments. The assay offers a sensitivity of 74 pM of UO_2_^2+^ by visual inspection and 7 pM by UV–visible spectrophotometer, both of which are far below 130 nM, the maximum UO_2_^2+^ concentration allowed in drinking water by the U.S. Environmental Protection Agency (Washington, DC, USA). 

## 4. Peroxidase-Mimicking DNAzyme (G4-DNAzyme) as Colorimetric Signal Transducer

**Figure 8:**
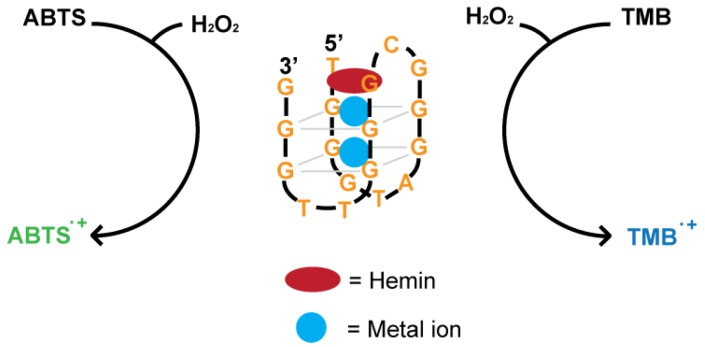
Proposed structure of the 18-nucleotide G4-DNAzyme (**middle**). The DNAzyme can catalyze oxidation of ABTS (**left**) or TMB (**right**) to enable a color change.

As discussed earlier, DNAzymes hold specific advantages over protein enzymes, particularly in biosensor development. Given the utility and advantages of peroxidases, increasing attention has been given to the development of artificial DNA enzymes mimicking peroxidase function. A DNAzyme with peroxidase-like activity was first reported by Sen and co-workers in the late 1990s [85,86,87]. Two G-rich DNA aptamers (PS2.M and PS5.M) were found to display selectivity towards *N*-methyl mesoporphyrin IX (NMM) and hemin. Later, it was revealed that these aptamers could act as DNAzymes to catalyze porphyrin metallation. Compared to free hemin, PS2.M also had 250-fold enhanced peroxidase activity. The proposed secondary structure of PS2.M with hemin is shown in Figure 8. In such a DNAzyme, each guanine is linked with neighboring guanine via two hydrogen bonds by Hoogsteen pairing. These cyclic guanine quartets then stack on each other in a helical fashion, forming a G-quadruplex structure. Hemin, with its Fe^III^-centered core, stacks externally on the terminal G-quartet through strong π-π interactions. The proximity between the oxygen atoms of guanine and Fe^III^ allows for this interaction, which is essential for peroxidation activity [88].

G-quadruplexes (G4) can be classified in terms of the stoichiometric as unimolecular, bimolecular, trimolecular and tetramolecular, and also in terms of orientation as parallel, antiparallel or mixed. The structure of G4 depends on the composition and length of the DNA, the orientation of the chains and positions of the loops, and also on the nature of the cations [89,90,91,92,93]. It has been shown that the parallel structure may be favored over the antiparallel [94,95,96]. DNA with longer loops generate antiparallel quadruplexes whereas shorter loops may favor parallel quadruplexes, minimizing steric hindrance [94]. The addition of potassium ions (K^+^) was reported to change antiparallel G4 configurations to parallel or hybrid G4 arrangements [95].

The catalytic activity of G4-DNAzymes largely depends on the structure of G4. The highest peroxidase activity was observed for parallel or mixed intramolecular quadruplexes [96,97]. This is related to their ability to bind hemin. Hemin binds with external guanines in a quadruplex, which indicates that parallel structure is more favorable for the binding of ligands through end-stacking. Lower activity of antiparallel quadruplexes is likely connected with steric hindrance caused by loops, which makes hemin binding difficult [98]. Yao and coworkers recently noticed a unique intramolecular enhancement effect of the adjacent adenine at 3′ end of G4 core sequences that significantly improves the activity of G4-DNAzymes [99]. Cations are crucial for the quadruplex formation and catalytic activity. Early studies indicated that the presence of potassium ion (K^+^) was necessary for quadruplex formation [85]. However, a recent report showed that other ions could also be used and that, in some cases, replacing K^+^ with NH_4_^+^ ions positively influenced enzymatic activity [100]. The peroxidase activity of DNAzymes was reported to depend on H_2_O_2_ concentration, but not on substrate concentration, indicating a different reaction mechanism for DNAzymes compared to HRP whose activity increases with substrate concentration [88]. A recent study showed that G4-DNAzymes also exhibit broader substrate selection when compared to HRP [101].

Visual detection can be achieved using 2,2′-azino-bis (3-ethylbenzothiazoline-6-sulfonic acid) (ABTS) as the substrate, which is converted to a green soluble product in the presence of H_2_O_2_ upon oxidation. Shen and co-workers also noticed that the addition of ATP had a positive effect on enzymatic activity in the reaction with ABTS [96]. ATP inhibits disproportion of ${\mathrm{ABTS}}^{⚫+}$, making ABTS a great substrate for a peroxidase-like reaction. TMB is an alternative chromogenic substrate, producing a blue-colored product upon oxidation.

In comparison with HRP, G4-DNAzymes possess numerous advantages including small size, ease of synthesis, as well as facile manipulation and amenability to the rational design of allosteric control. G4-DNAzymes have been extensively exploited to replace HRP as the colorimetric signal transducer for the development of colorimetric biosensors and molecular machines. In this section, we will discuss the signaling strategies of colorimetric biosensors using G4-DNAzymes. 

### 4.1. Metal Ion-Mediated Signaling of G4-DNAzyme

The formation and catalytic activity of G4-DNAzymes largely rely on metal ions. As a result, G4-DNAzymes can be used to optically sense metal ions or metal ion-associated targets.

Wang and co-workers postulated that G4-DNAzyme’s catalytic activity could be selectively induced by K^+^ [102]. They proved the assumption of K^+^ selectivity by performing comparative tests with other ions such as Na^+^, Li^+^, NH_4_^+^, Cs^+^, Mg^2+^ and Ca^2+^ using TMB as an indicator for DNAzyme activity. This reaction enabled visualization of K^+^ ions to the naked eye. The lowest detection limit achieved for K^+^ ions was 2 μM. This assay was also used for the detection of K^+^ ions in real-life samples, such as human serum. Li and Zhang developed a more sensitive detection assay for K^+^ ions by using G4 complex with single-walled carbon nanotubes (SWNTs) [103]. They found that free hemin, which can cause a high background signal, could be strongly adsorbed on the surface of SWNTs. Therefore, SWNTs can be used to decrease the background signal of a G4-hemin DNAzyme sensing platform, which allowed for the colorimetric detection of potassium to a concentration of 2 nM. 

**Figure 9:**
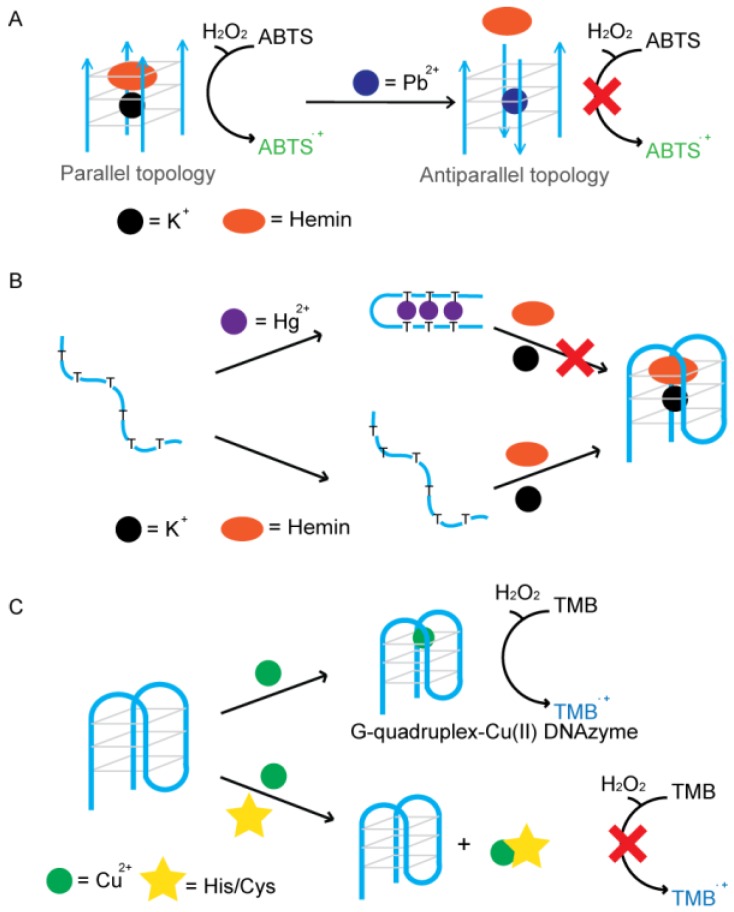
G4-DNAzyme-based biosensors mediated by metal ions. (**A**) Using potassium-lead-switched G4-DNAzyme for Pb^2+^ detection; (**B**) Colorimetric detection of aqueous Hg^2+^ using Hg^2+^-modulated G4-DNAzyme; (**C**) Colorimetric detection of histidine and cysteine based on G4-Cu(II) DNAzyme.

Metal ions, such as Pb^2+^, can induce a parallel-to-antiparallel conformational change of a potassium stabilized G4-DNAzymes (PW17) and inhibit its peroxidase activity. Based on this feature, an assay for the detection of Pb^2+^ was presented by the Dong group (Figure 9A) [104,105]. A decrease in the catalytic activity of DNAzymes was proportional to the concentration of lead added to the sample containing K^+^ ions. This allows for quantitative analysis of aqueous Pb^2+^ using the ABTS-H_2_O_2_ colorimetric system. The lowest detection limit for Pb^2+^ was 32 nM. Sodium (Na^+^) is another metal ion that can affect the catalytic activity of potassium stabilized G4-DNAzyme. Tang and co-workers reported that p25 G4-DNAzyme exhibits a conformational transition from hybrid-type to antiparallel with increasing concentrations of Na^+^ in the presence of K^+^, resulting in a decrease of the p25-hemin DNAzyme activity [106]. A colorimetric probe of Na^+^ is thus designed with a detection limit of 0.6 mM.

Mercury ions (Hg^2+^) are able to specifically bind to the thymine-thymine (T-T) mismatch in a DNA duplex. This binding has also been used in folding ssDNA into dsDNA, for strengthening DNA duplexes and for activating DNAzymes and DNA-based machines. Wang and co-workers designed an assay that allowed for the colorimetric detection of mercury down to a concentration of 50 nM (Figure 9B) [107]. They used oligonucleotides rich in thymine residues that could form quadruplex structures with catalytic activity. However, after the addition of mercury ions, T–Hg^2+^–T base pairs destabilized the quadruplex structure and inhibited enzymatic activity. The Shen group proposed a “turn on” sensor for which the catalytic activity is observed in the presence of Hg^2+^ [108]. Thymine-containing residues were added to the 5′ ends of oligonucleotides that can form intermolecular quadruplexes. In the presence of mercury, T–Hg^2+^–T base pairs were formed which facilitated the formation of G4 leading to the increase of catalytic activity. The detection limit for this design was 19 nM.

Similarly, given that silver ions (Ag^+^) can stabilize cytosine-cytosine (C–C) mismatches by forming C–Ag^+^–C base pairs, Shen and co-workers presented an assay for Ag^+^ using G4-DNAzyme which allows the detection of aqueous Ag^+^ at concentrations as low as 6.3 nM [109]. The same group also took advantage of the strong bond between Ag^+^ and cysteine for the detection of cysteine. Cysteine broke C–Ag^+^–C bonds leading to the reformation of the DNA duplex and reduced catalytic activity of the system [110]. This method allows the colorimetric detection of cysteine with a detection limit of 25 nM.

Recently, Wang and coworkers proposed a colorimetric strategy for simultaneous detection of histidine and cysteine based on G4-Cu(II) DNAzyme (Figure 9C) [111]. As strong binders with Cu^2+^, histidine and cysteine can disturb the formation of G4-Cu(II) DNAzyme complex, thus leading to a low catalytic activity. With this strategy, the limit of detection in experimental measurement for histidine and cysteine was 10 nM and 5 nM, respectively. With the help of *N*-ethylmaleimide, cysteine is alkylated and the reaction between Cu^2+^ is inhibited, so selectivity can also be established.

### 4.2. DNA-Mediated Signaling of G4-DNAzyme

Being nucleic acids permits hybridization of single-stranded G4 DNA molecule to its complementary sequence (blocker DNA) that prevents the formation of the G-quadruplex structure and inhibits its catalytic ability. Various colorimetric biosensors have been developed based on target-dependent removal of this blocker DNA.

**Figure 10:**
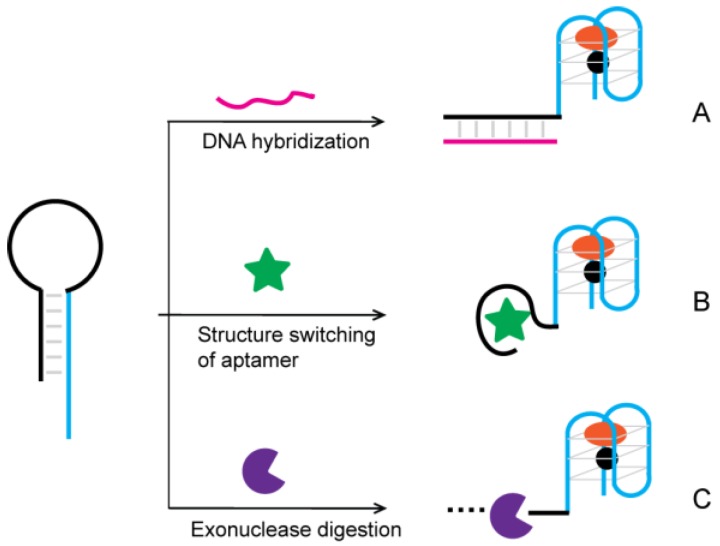
G4-DNAzyme-based biosensors signaling though removal of blocker DNA. Hairpin can be opened through: (**A**) DNA hybridization; (**B**) structure switching of an aptamer; (**C**) exonuclease-mediated digestion.

Blocker DNA (bDNA) could be released through DNA hybridization (Figure 10A). Willner and coworkers [112] designed the first G4-DNAzyme containing a hairpin structure, whose duplex structure at the stem prohibited the self-assembly. Following target DNA hybridization, the loop region opens and leads to the formation of a hemin/G-quadruplex structure that could oxidize ABTS to the colored ${\mathrm{ABTS}}^{⚫+}$. This assay allows colorimetric detection of DNA at a concentration of 0.2 µM. This colorimetric strategy was further extended to the detection of telomerase activity in cancer cells with a detection limit of 500 cells [112]. Yang and colleagues reported a strategy making use of nicking endonuclease (NEase) to improve the sensitivity of the DNA assay [113]. Upon hybridization with a target, the NEase recognizes a specific nucleotide sequence and cleaves the hairpin-DNAzyme probe into two fragments resulting in the dissociation of the target DNA from the fragments of the hairpin. Amplification is accomplished by another hairpin-DNAzyme probe hybridizing to the released intact target to continue the strand-scission cycle, leading to the activation of numerous DNAzymes. The detection limit of the colorimetric method was 10 pM.

The bDNA can also be part of an aptamer. In the presence of its target ligand, the formation of the respective ligand–aptamer complex would open up the hairpins and result in the self-assembly of the activated G4-DNAzyme (Figure 10B). Willner and coworkers demonstrated this concept by using an AMP aptamer and a lysozyme aptamer [114]. A detection limit of 50 µM and 0.5 pM was reported for analyzing AMP and lysozyme, respectively. Several other sensors were also reported that were based on the same principle but used different aptamers, such as ATP [115], methamphetamine [116], and Ochratoxin A [117].

The bDNA could also be digested by exonucleases (Figure 10C). Yu and coworkers describe a colorimetric strategy for estimating the activity of polynucleotide kinase (PNK) by taking advantage of the efficient cleavage of exonuclease and the G4-DNAzyme signal amplification [118]. The 5′-OH of the hairpin DNA was first phosphorylated in the presence of PNK and then digested by an exonuclease. As a result, the blocked G4-DNAzyme sequence was released due to the removal of its completely complementary sequence. Because of the complete blocking and efficient releasing of G4-DNAzyme, the colorimetric method exhibited an excellent performance in PNK analysis with a low detection limit of 0.06 U/mL and a wide detection range from 0.06 to 100 U/mL. Tang and coworkers reported a strategy that applied G4-DNAzyme-containing hairpin into polymerase chain reaction (PCR) [119,120]. During the annealing step of PCR amplification, the hairpin DNA probe would open and form a stable duplex with one single-stranded PCR product. Taq DNA polymerase has an inherent 5′-3′ exonuclease activity and cleaves a part of the probe that is hybridized to the PCR template and releases the G4-DNAzyme. After PCR amplification, the G4-DNAzyme can produce a green color in the presence of hemin, ABTS, and H_2_O_2_. Detection of as low as 10 copies of HBV DNA was achieved based on this method.

### 4.3. Signaling by Reconstructing Split Fragments of G4-DNAzyme

**Figure 11:**
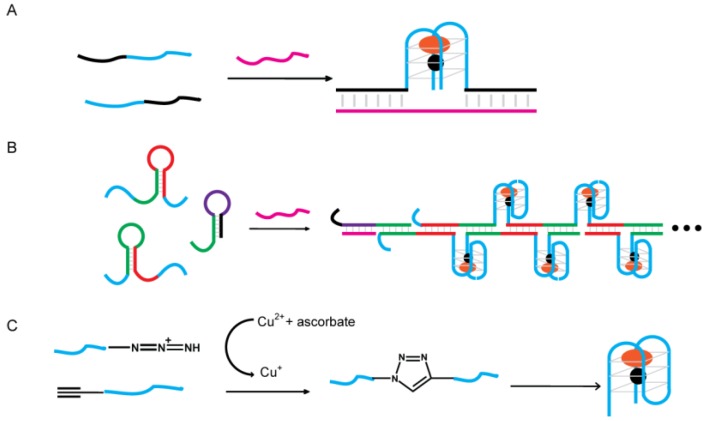
Biosensors signaling by reconstructing split fragments of G4-DNAzyme. (**A**) Reconstructing G4-DNAzyme by using target DNA as template; (**B**) Scheme for the amplified detection of DNA through the enzyme-free autonomous assembly of G40 DNAzyme Nanowires; (**C**) Colorimetric detection of Cu^2+^ using click chemistry and G4-DNAzyme.

G4-DNAzyme would lose its peroxidase activity if the G-quadruplex sequence was split into two halves, but once it is reconstructed with the assistance of designated template, the catalytic activity can be restored. Kolpaschikov demonstrated this concept by using a single-stranded synthetic DNA as the template for two equally split (1:1) G4-DNAzyme sequences (Figure 11A). This design was further applied to achieve visual single nucleotide polymorphism typing [121]. Zhou and co-workers later showed that the asymmetric 3:1 split of G4-DNAzyme worked better than the 1:1 split [122]. Tao and colleagues presented a three-way junction-based system for gene transcript detection [123]. Only upon the introduction of the target gene transcript offering a specific recognizable splicing site did the two probes assemble into the three-way junction arrangement, thus providing a functional G4-DNAzyme. The detection limit was 0.063 μM. Similar designs have also been applied for detecting PCR products [124] and thrombin [125].

Willner and coworkers presented an amplification platform that involves the use of split fragments of G4-DNAzyme that upon recognition of the target DNA, can trigger an autonomous cross-opening process that yields the formation of hemin/G4 DNAzyme wires (Figure 11B) [126]. The resulting nanowires then enable colorimetric or chemiluminescent readout of the sensing process. This analytical platform allows the sensing of target DNA with a detection limit of 0.1 pM. This amplification platform has also been used in detecting Hg^2+^ [127] and microRNA [128] with a detection limit of 9.7 pM and 7.4 fM, respectively.

An alternative method to reunite the split fragments of G4-DNAzyme is ligation. Zeng and coworkers constructed a colorimetric biosensor for rapid detection of Cu^2+^ (Figure 11C) [129]. The reaction mechanism is based on the formation of a G4-DNAzyme using Cu^2+^-promoted click chemistry between azide- and alkyne-modified short G-rich sequences, followed by the self-assembly of hemin/G-quadruplex DNAzyme with the aid of hemin and K^+^. The visual detection limit of Cu^2+^ with this system was 100 nM. 

### 4.4. Signaling of G4-DNAzyme by Nucleotide-Cleaving DNAzymes

**Figure 12:**
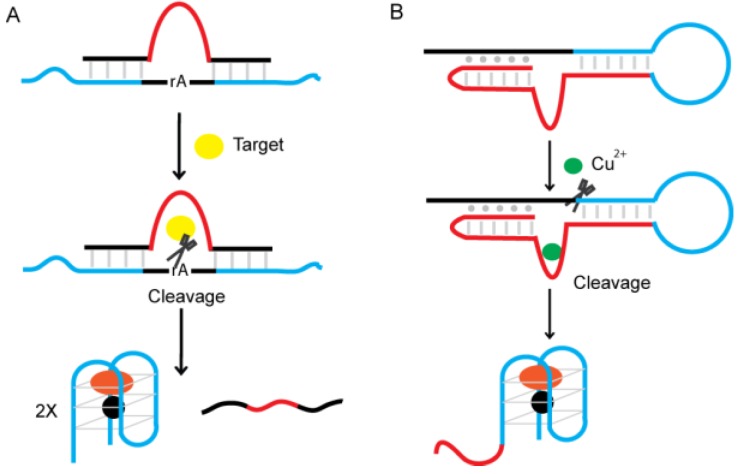
Design of dual-DNAzyme probes using G4-DNAzyme and (**A**) an RNA-cleaving DNAzyme; or (**B**) a Cu^2+^-specific DNA-cleaving DNAzyme.

Dual-DNAzyme sensors have also been developed. Willner and coworkers described a supermolecular construct which comprised of target-dependent RNA-cleaving DNAzymes, their substrate sequences, and G4-DNAzymes [130], as shown in Figure 12A. The DNAzyme is hybridized to their RNA substrate sequence that includes a G4-DNAzyme on each end of the substrate sequence. The partial hybridization between G4-DNAzyme domains and RNA-cleaving DNAzyme prohibits the self-assembly of the G-quadruplex. In the presence of target, the DNAzyme cleaves the RNA, fragmenting the sequence into two parts, which destabilize the complex and enable the formation of an activated G4-DNAzyme. By making use of this strategy, the Willner group successfully developed colorimetric assays that can sensitively and specifically detect Pb^2+^, l-histidine, UO_2_^2+^ or Mg^2+^ [130,131]. They further proved that the constructs can be applied to perform the “AND” and “OR” logic gate operations [131].

Xie and co-workers [132] proposed a dual-DNAzyme unimolecular probe for the colorimetric detection of Cu^2+^ (Figure 12B). This assay utilizes an oligonucleotide composed of three segments: a DNA substrate domain, the G4-DNAzyme domain, and a Cu^2+^-dependent DNA-cleaving DNAzyme domain. As a result of strong intramolecular interactions in the absence of Cu^2+^ ions, an internal structure is generated that prevents the formation of the quadruplexes. In the presence of Cu^2+^, the DNA-cleaving DNAzyme is activated and cleaves the DNA substrate. After releasing the middle domain, the active G4-DNAzyme is formed. Changes in absorbance (from the reaction of TMB with H_2_O_2_) are proportional to copper ion concentration. The detection limit of this method is 1 µM.

### 4.5. Signaling by Synthesis of G4-DNAzymes Using Polymerase

**Figure 13:**
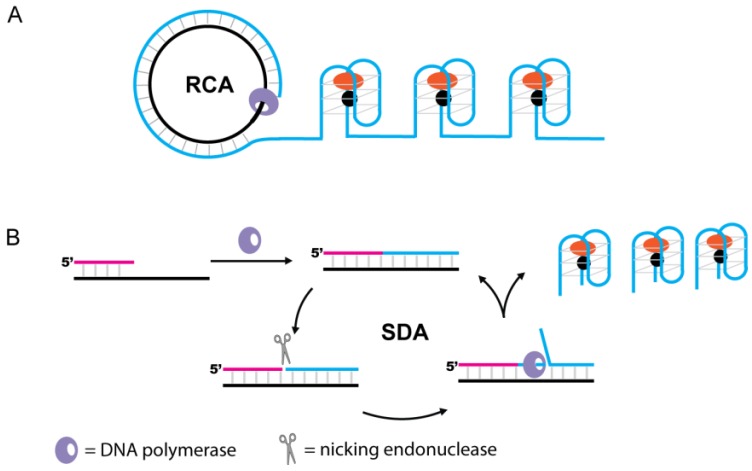
G4-DNAzyme-based biosensors signaling by polymerase-stimulated DNAzyme synthesis. (**A**) Synthesis of G4-DNAzymes using RCA; (**B**) Synthesis of G4-DNAzymes using SDA.

As G4-DNAzymes are simply DNA molecules, DNA polymerases can be used in various biosensing platforms to generate G4-DNAzymes. Polymerase-stimulated synthesis, such as rolling circle amplification (RCA) [133,134] or strand displacement amplification (SDA) reaction [135], to produce G4-DNAzymes was adopted by many researchers to develop analytical methods with high sensitivity (see Figure 13). These methods do not require thermal replication cycles, costly optical labels, or dedicated instrumentation [136,137]. Upon recognition of the input analyte, the amplification reaction is activated and numerous G4-DNAzymes were synthesized, in turn generating colorimetric signals. Thus, a single recognition event can be translated into an amplified signal.

### 4.6. Signaling by Solid Support-Mediated Separation of G4-DNAzyme

**Figure 14:**
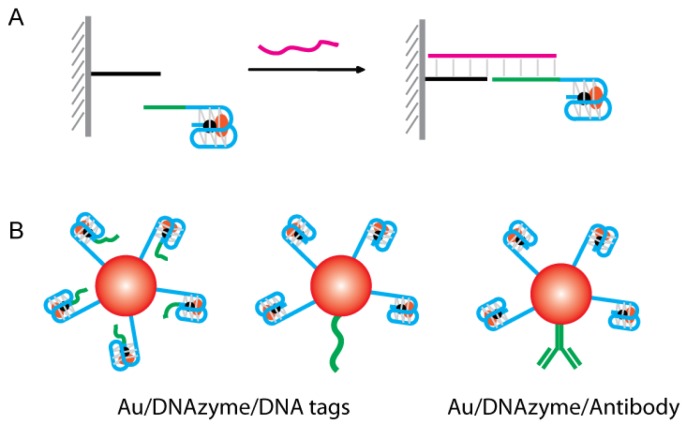
G4-DNAzyme-based biosensors signaling by solid support-mediated separation. (**A**) Target DNA-mediated G4-DNAzyme immobilization; (**B**) “Bio-bar-code” amplification.

DNA detection can also be achieved using target DNA as a linker to bridge surface-immobilized DNA with free-floating G4-DNAzymes (Figure 14A). The signal could be further enhanced by using AuNPs immobilized with G4-DNAzymes (Figure 14B). AuNPs possess a very high surface-to-volume ratio, which offers an opportunity to attach multiple kinds of biomolecules, such as aptamers, DNAzymes and antibodies, as multifunctional nanoprobes [138]. When AuNPs were used as carriers for the G4-DNAzyme labels, a single recognition event could be converted to multi-labels of G4-DNAzymes, significantly improving the sensitivity. This kind of signal amplification has been named “bio-bar-code” amplification. G4-DNAzyme-functionalized AuNPs were used as biocatalytic conjugates by Park and coworkers for the construction of colorimetric sensors for the amplified detection of chlamydia gene [139]. The detection limit was 50 fM. Similar designs have also been reported for the detection of *invA* gene of *Salmonella* [140], thrombin [141], ATP [142], and myoglobin [143].

## 5. Organic Dyes as Colorimetric Signal Transducer

Many color-based biotechnologies often rely on natural or synthetic dyes. Through the years, synthetic dyes have become useful for not only their pigmentation, but also their physical and chemical properties that can be engineered to induce a color change. In the field of biosensors, dyes are designed to play the role of colorimetric signal transducers when using DNAzymes as recognition elements.

### 5.1. Signaling by DNA-Binding Dyes

**Figure 15:**
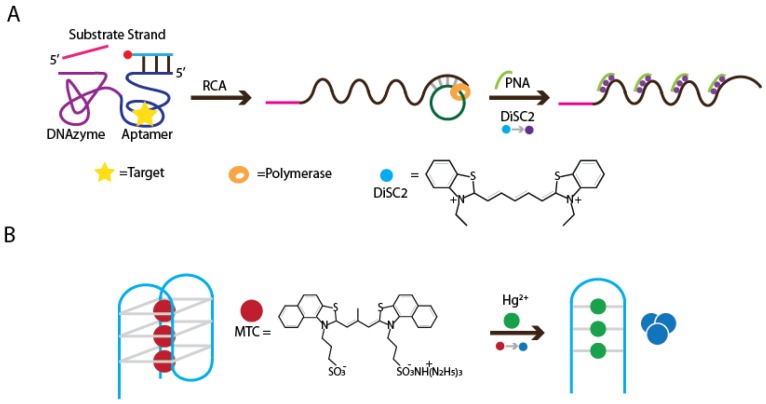
Colorimetric detection using organic dyes. (**A**) Colorimetric detection involving an RNA-cleaving DNAzyme for RCA initiation and signaling by DiSC2(5); (**B**) MTC-based detection through Hg^2+^-induced structure change from G-quadruplex to duplex.

Colorimetric assays can be developed from duplex-binding dyes, such as DiSC2(5) (3,3′-diethylthiadicarbocyanine). Our group has developed a method that utilized RNA-cleaving DNAzymes, RCA, and DiSC2(5) [144]. The recognition element is an allosteric DNAzyme whose RNA-cleaving activity is regulated by an aptamer. Target binding to the aptamer induces a conformation change, enabling the allosteric DNAzyme to cleave its RNA-containing substrate, and generating a DNA primer. Φ29 DNA polymerase uses the DNA primer to initiate an RCA reaction, resulting in the production of a long DNA chain with tandem repeats that can bind a peptide nucleic acid (PNA). The PNA hybridizes to the RCA product to form highly stable duplex structures that can bind DiSC2(5). When bound, DiSC2(5) undergoes color change from blue to purple, indicating the presence of a target (Figure 15A). Signal amplification offered by RCA is able to provide a detection limit of 100 µM when an ATP target was tested.

Another study done by Chen and coworkers explored the use of cyanine dye as a colorimetric reporter of Hg^2+^-induced conformational change in G-quadruplex structures [145]. Traditionally, G-quadruplex visual probes relied on catalysis of H_2_O_2_-mediated oxidation reaction to cause color change. However, oxidation reactions can be easily perturbed by environmental and temporal factors. To achieve better stability, 3,3′-di(3-sulfopropyl)-4,5,4′,5′-dibenzo-9-methylthiacarbocyanine triethylammonium salt (MTC) supramolecule was used for the signal production process (Figure 15B). In contaminated samples, Hg^2+^ binds to thymine residues of a G4-DNAzyme, causing the G-quadruplex to become a duplex. MTC supramolecules assume a red monomer form when bound to G-quadruplex, and dissociates from duplex DNA to form blue aggregates. This color change can be observed by the naked eye for Hg^2+^ concentration down to 1 μM. For superior sensitivity, Hg^2+^-induced motif transition and the subsequent loss of MTC-DNA interaction are accompanied by a sharp decrease in absorption at 656 nm and an increase at 580 nm and, therefore, this method can also achieve quantitative detection of Hg^2+^. 

### 5.2. Signaling by pH Indicators

**Figure 16:**
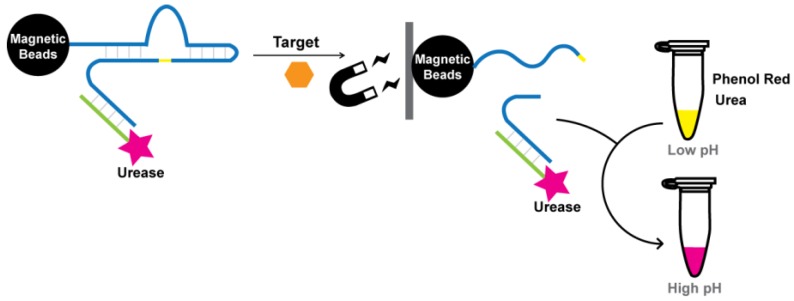
Conceptual schematic of target-induced DNAzyme cleavage and consequent release of urease into solution. Signal is transduced through hydrolysis of urea and reported through color change of phenol red.

In addition to DNA-binding dyes, pH indicators provide another platform for colorimetric detection. Our group coupled urease with an RNA-cleaving DNAzyme to create a modified litmus test for *E. coli* [146,147]. The assay involved a DNAzyme with a 5′ biotin moiety for binding to streptavidin-coated magnetic beads (MB) and a 3′ end for hybridization with urease-conjugated DNA (UrDNA). To prepare UrDNA, urease was attached to a 5′-amino-modified DNA oligonucleotide using 3-maleimidobenzoic acid *N*-hydroxysuccinimide ester. In the presence of *E. coli*, the DNAzyme catalyzes cleavage at a ribonucleic site along the sequence, releasing the 3′ end of DNAzyme and its bounded UrDNA into solution. After removal of the MB-immobilized cleavage product via magnetic separation, urea was added to the solution. Urease catalyzes the hydrolysis of urea into ammonia, causing an increase in pH that can be translated into a color change using phenol red (Figure 16). Within one hour of substrate addition, a sharp color shift from yellow to pink was observed for a sample containing 5 × 10^5^ *E. coli* cells.

## 6. Conclusions

Since the emergence of the glucose meter, the field of biosensors has expanded tremendously. The development of the in vitro selection technique has allowed for the discovery of a great range of DNAzymes over last 20 years, with excellent ability in both target recognition and enzymatic catalysis. DNAzymes have shown great potential to serve as target recognition elements and replace antibodies in biosensor development. The substantial development in DNAzyme-based colorimetric biosensors, as spotlighted in this review, further validates the idea that DNAzymes can be easily integrated with various colorimetric signal transduction elements, such as AuNPs, HRP, and organic dyes, to achieve practically useful applications. We have also witnessed the wide use of G4-DNAzyme in place of enzyme horseradish peroxidase (HRP) for color generation, which also nicely showcases the neat role of DNAzymes in signal transduction for biosensor development.

With the increasing demand for point-of-care diagnostics in medical practices and simple devices for either environmental monitoring or consumer-oriented applications, research and development on simple biosensors will continue to flourish in coming years. DNAzymes, and functional nucleic acids in general, will continue to receive great attention in the biosensing research community as they are much more stable alternatives and, in principle, can be made for any target of choice. As small DNA oligonucleotides, they are relatively cheap to produce and widely available. However, most of the research at the current stage has focused on proof-of-concept demonstrations of novel sensor designs using a few well-studied DNAzymes that are activated by metal-ion cofactors such as Pb^2+^ and Cu^2+^. More efforts are certainly needed to develop diversified DNAzymes for other important analytes and biomarkers. In recent years, there have been noticeable activities in developing DNAzymes for a wide range of metal ions [148,149,150,151], bacterial pathogens [152,153,154] and specific cancer cells [155]. We believe that these and future DNAzymes can be coupled with the colorimetric biosensor designs described in this review to achieve a wide range of applications.

## Figures and Tables

**Table 1:** Techniques for AuNPs’ Dispersal and Aggregation.

Techniques	Reference
Colloidal Stabilizers	[45,46,47,48,49,50,51]
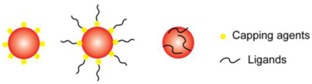	
The addition of surface-tethered capping agents (e.g., thiols, amines, phosphines) and ligands (e.g., small-charged species, macromolecules, polymers), on the surface of AuNPs, can enhance the stability of colloid AuNPs.	
High Salt Concentration	[52,53,54,55]
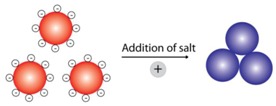	
High salt concentrations reduce colloid stability via increase of electrostatic interaction. This will result in the aggregation of AuNPs, and a color shift from red to blue will be observed.	
Inter-Particle Cross-Linking	[56,57,58]
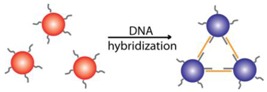	
When the surface of AuNPs is modified with ssDNA, AuNPs can be bridged through DNA hybridization. This can lead to the aggregation of AuNPs and a color shift from red to blue can be observed.	
